# Iron Sulfide Enhanced the Dechlorination of Trichloroethene by *Dehalococcoides mccartyi* Strain 195

**DOI:** 10.3389/fmicb.2021.665281

**Published:** 2021-06-01

**Authors:** Yaru Li, He-Ping Zhao, Lizhong Zhu

**Affiliations:** ^1^College of Environmental and Resource Sciences, Zhejiang University, Hangzhou, China; ^2^Key Laboratory of Organic Pollution Process and Control, Zhejiang University, Hangzhou, China

**Keywords:** *Dehalococcoides mccartyi* strain 195, FeS, trichloroethene, electron transport, iron-sulfur cluster

## Abstract

Iron sulfide (FeS) nanoparticles have great potential in environmental remediation. Using the representative species *Dehalococcoides mccartyi* strain 195 (*Dhc* 195), the effect of FeS on trichloroethene (TCE) dechlorination was studied with hydrogen and acetate as the electron donor and carbon source, respectively. With the addition of 0.2 mM Fe^2+^ and S^2–^, the dechlorination rate of TCE was enhanced from 25.46 ± 1.15 to 37.84 ± 1.89 μmol⋅L^–1^⋅day^–1^ by the *in situ* formed FeS nanoparticles, as revealed through X-ray diffraction. Comparing the *tceA* gene copy numbers between with FeS and without FeS, real-time polymerase chain reaction (PCR) indicated that the abundance of the *tceA* gene increased from (2.83 ± 0.13) × 10^7^ to (4.27 ± 0.21) × 10^8^ copies/ml on day 12. The transcriptional activity of key genes involved in the electron transport chain was upregulated after the addition of FeS, including those responsible for the iron–sulfur cluster assembly protein gene (DET1632) and transmembrane transport of iron (DET1503, DET0685), cobalamin (DET0685, DET1139), and molybdenum (DET1161) genes. Meanwhile, the reverse transcription of *tceA* was increased approximately five times on the 12th day. These upregulations together suggested that the electron transport of *D. mccartyi* strain 195 was enhanced by FeS for apparent TCE dechlorination. Overall, the present study provided an eco-friendly and effective method to achieve high remediation efficiency for organohalide-polluted groundwater and soil.

## Introduction

Trichloroethene (TCE) has been extensively used as a solvent in pesticides, dry cleaning, and anesthesia medicine (Vamvakas et al., 1998; Chowdhury and Viraraghavan, 2009). It has become one of the most detected organohalides in subsurface water because of improper disposal and leakage. Classified as a carcinogen (group I), TCE poses a threat to human respiratory organs and even human life (Stewart, 2001; Guha et al., 2012). Microbial reduction is a promising technique for TCE remediation. In particular, *Dehalococcoides* members can completely dechlorinate TCE to the benign product ethene (Maymo’-Gatell et al., 1997; Antoniou et al., 2019; Zhao and He, 2019; Wang et al., 2020).

The respiratory dechlorination of TCE can yield energy for the growth of *Dehalococcoides*, in which electrons are transferred from hydrogen to organohalides *via* the membrane-associated electron transport chain (ETC) (Magnuson et al., 2000; Löffler et al., 2013). Because of the lack of genes for the synthesis of quinones and cytochromes, *Dehalococcoides* uses the complex iron–sulfur molybdoenzyme (CISM) complex to transfer electrons from hydrogenase to reductive dehalogenase (RDase) (Löffler et al., 2013; Kublik et al., 2016). The major components of ETC include hydrogenases, CISM, and RDases (Seshadri et al., 2005; Pinske et al., 2011; Hartwig et al., 2017). The dominant hydrogenase Hup decomposes hydrogen to electrons and transfers these electrons to CISM using the iron–sulfur cluster subunit (Seshadri et al., 2005; Türkowsky et al., 2018). The CISM complex was formerly annotated as formate dehydrogenase containing multiple iron–sulfur clusters for electron transport (Wang et al., 2018). These iron–sulfur clusters are cubic crystal structures [4Fe-4S] and may have a low redox potential. Compared with quinone proteins, the lower redox potential of iron–sulfur clusters could avoid endothermic reactions, such as the reverse transfer of electrons (Löffler et al., 2013; Kublik et al., 2016). However, the accurate molecular components of the CISM complex and its exact functions are still unclear. The terminal electron sink of ETC is RDase, which also contains iron–sulfur clusters to accept electrons from the CISM complex (Schipp et al., 2013; Schubert et al., 2018). RDase is encoded by the *tceA* gene and catalyzes TCE dechlorination to vinyl chloride (VC) (Mansfeldt et al., 2014). From the ETC components, iron–sulfur clusters are crucial for organohalide reduction throughout the ETC, and close proximity ensures rapid intraprotein or interprotein electron transfer (Costentin et al., 2005; Payne et al., 2015).

Iron sulfide (FeS) has also been studied extensively for the abiotic degradation of chlorinated ethenes (Jeong and Hayes, 2007; He et al., 2015). With a high concentration of FeS (>10 g/L), the degradation products of TCE by FeS vary, including acetylene, *cis-*dichloroethene (*cis-*DCE), 1,1-DCE, and VC, under weakly alkaline conditions (Liang et al., 2007; He et al., 2010). However, the dechlorination rate of chlorinated ethenes by FeS was slow compared with biotic dechlorination. Thus, the interaction between FeS and microbes was studied. On the one hand, FeS acts as a naturally occurring electrical wire, bridges spatially discrete environments, and mediates long-distance extracellular electron transfer (Kondo et al., 2015); on the other hand, FeS can enter the periplasm of sulfate-reducing bacteria to accelerate electron transport (Deng et al., 2020). Moreover, the composition of FeS is the same as that of the iron–sulfur clusters. Thus, we hypothesized that FeS nanoparticles could enhance electron transport in *Dehalococcoides*.

Using the representative species *Dehalococcoides mccartyi* strain 195 (*Dhc* 195), we investigated the effects of FeS on both TCE dechlorination and the growth of *Dhc* 195. The underlying mechanism was explored by X-ray diffraction and transcriptomic analysis. Overall, this study provided an eco-friendly and effective way to improve the bioremediation of organohalides.

## Materials and Methods

### Materials

TCE (99.5%), *cis-*DCE (98%), and VC (99%) were purchased from J&K Chemicals (Shanghai, China). FeCl_2_⋅4H_2_O and Na_2_S were purchased from Aladdin (Shanghai, China). All other chemicals were of analytical reagent or guaranteed reagent grade.

### Culture and Growth Conditions

*Dhc* 195 was donated by Jun Yan from the Institute of Applied Ecology, Chinese Academy of Sciences. *Dhc* 195 was cultivated in a defined mineral salt medium with 10 mM acetate as the carbon source and 2.5 mM H_2_ as the electron donor. The mineral salt medium was described by Löffler et al. (2005). Briefly, 10 ml of salt solution (specific components not shown), 1 ml of Se/W solution, 1 ml of trace element solution, 0.25 ml of 0.1% (w/v) resazurin stock solution, and 2.292 g of N-[Tris(hydroxymethyl) methyl]-2-aminoethanesulfonic acid were added to 1 L of double-distilled water. The medium was boiled and flushed with N_2_ to remove oxygen. Meanwhile, 0.242 g of L-cysteine, 0.0771 g of DL-dithiothreitol, and 2.52 g of NaHCO_3_ were added as reducing agents and buffer, respectively. Then, the medium was transferred into glass serum bottles sealed with butyl rubber stoppers and aluminum crimps. The medium was autoclaved and stored at 30°C. Before each experiment, 1 ml of ATCC vitamin supplement (ATCC MD-VS, United States) and 5 mL of the inoculum culture were transferred into one bottle, and the next steps were the same as described by Li et al. (2019). All cultures were incubated at 30°C in the dark.

### Batch Experiments

Batch experiments were performed in 100 mL serum bottles with 50 mL of anaerobic medium (pH ≈ 7.2). Solid-state FeCl_2_⋅4H_2_O and Na_2_S⋅9H_2_O were purchased from Aladdin (United States). Eighty milligrams of FeCl_2_⋅4H_2_O and 96 mg of Na_2_S⋅9H_2_O were dissolved in 20 ml of anaerobic water and added to serum bottles with injectors in an anaerobic glove box. Then, Fe^2+^ and S^2–^ formed black nanoparticles immediately. In all experiments, the sealed bottles were rotated at 200 rpm and 30°C in the dark. All the experimental results are presented as the average values from triplicates (error bars are shown in the associated figures).

### Chemical Analysis

#### Characterization of Chloroethenes

Chloroethenes and ethene were detected by a gas chromatography-flame ionization detector (Agilent Technologies GC system, model 6890N, Agilent Technologies Inc., United States) equipped with a packed column (30 m long, 0.32 mm i.d., 0.5 mM thickness, cross-linked polydimethysiloxane film, J&W Scientific, United States). The detection method was described by Wen et al. (2015, 2020) using 100-μL headspace samples. The oven was kept at 60°C for 2 min, heated gradually to 120°C (20°C/min), and finally kept at 120°C for 2 min. The concentration of chloroethenes was calculated based on gas–liquid equilibrium using Henry’s law constants. The Henry’s law constants of TCE, *cis-*DCE, VC, and ethene were 0.419, 0.167, 1.137, and 8.71, respectively.

#### Characterization of Iron Sulfide

The morphology of the formed iron–sulfur nanoparticles was characterized using scanning electron microscopy equipped with energy dispersive X-ray spectrometry (Zeiss Gemini 300, Germany). The crystal structure of iron–sulfur nanoparticles was characterized by X-ray diffraction (Bruker D8 Advance diffractometer, Germany) at the Analysis Center of Agrobiology and Environmental Sciences, Zhejiang University.

### Biological Analysis

#### Characterization of Cellular Morphology

The cellular morphology of *Dhc* 195 cells was characterized using transmission electron microscopy (JEM-1200EX, Japan). Liquid samples (50 mL) were centrifuged to collect cells, further immobilized by 2.5% glutaraldehyde and stored at 4°C for 12 h. After discarding glutaraldehyde, cells were washed three times with phosphate buffer (pH = 7.0). Osmic acid (1%) was used to immobilize cells again for 1.5 h, followed by three washes with phosphate buffer (pH = 7.0). Then, different concentrations of ethanol solution (30, 50, 70, 80, 90, 95, and 100%) were used for dehydration. Finally, acetone and embedding agent were used to treat cells.

#### DNA and RNA Extractions

Liquid samples (1.5 ml) obtained without concentrating the cells were centrifuged (21,000 × *g*, 10 min at 4°C) to collect cells. Chromosomal DNA was extracted using a DNeasy PowerSoil Pro Kit (QIAGEN, Germany) according to the manufacturer’s instructions. RNA was extracted using an E.Z.N. A Soil RNA Mini Kit (OMEGA, United States). Complementary DNA (cDNA) was synthesized using FastKing gDNA Dispelling RT SuperMix (TianGen Biotech, Beijing, China). The cDNA synthesis reaction was performed with an incubation time of 15 min at 42°C followed by 3 min at 95°C in a DNA Engine Peltier Thermal Cycler (Bio-Rad, CA, United States) (Doğan-Subaşı et al., 2014).

#### Real-Time Polymerase Chain Reaction

The dominant hydrogenase gene *hup*, formate dehydrogenase gene *fdh*, and RDase gene *tceA* were quantified. For *hup* and *fdh*, amplification was performed using a Step One Plus Real-Time Polymerase Chain Reaction (PCR) System (Applied Biosystems, United States) with SYBR green-based detection agents (Morris et al., 2006). The reaction solution contained 7.5 μL of SYBR Green mix (DBI Bestar, Ludwigshafen, Germany), 0.5 μL of Rox, 0.5 μL of each primer, 2 μL of DNA template, and 4 μL of double-distilled water. The program was run for 10 min at 95°C for Taq activation, followed by 40 cycles of 15 s at 95°C, 60 s at 55°C and a melting curve stage from 55 to 95°C. For the *tceA* gene, the method was the same as that described by Ritalahti et al. (2006). The method limit of quantitation was 10^3^ copies/mL. The sequences of primers and probes were synthesized by Sangon Biotech, as shown in Supplementary Table 1. The standard curves and amplification efficiency are shown in Supplementary Table 2.

#### Differential Gene Expression Assay

For the transcriptomic analysis, cultures were collected for RNA extraction when approximately 75% of TCE was dechlorinated. To collect sufficient material, 24 bottles of *Dhc* 195 without FeS and 24 bottles of *Dhc* 195 with FeS (0.2 mM) were inoculated and grown in triplicate. For each setup, cells were collected from eight bottles by centrifugation (21,000 × *g*, 10 min at 4°C). Then, RNA of two different setups was extracted using an E.Z.N.A. Soil RNA Mini Kit (OMEGA, United States). RNA quality was determined by a 2100 Bioanalyzer (Agilent) and quantified using an ND-2000 (NanoDrop Technologies). Only high-quality RNA samples (OD_260/280_ = 1.8∼2.0, OD_260/230_ ≥ 2.0, RIN ≥ 6.5, 28S:18S ≥ 1.0) were used to construct the sequencing library. A Ribo-Zero Magnetic Kit (epicenter) was used to remove ribosomal RNA, and then all messenger RNAs were broken into short fragments (200 bp) by adding fragmentation buffer. After that, a SuperScript double-stranded cDNA synthesis kit (Invitrogen, CA, United States) with random hexamer primers (Illumina) was used to synthesize double-stranded cDNA. Using Phusion DNA polymerase, PCR amplicons after 15 PCR cycles were sequenced with the Illumina HiSeq × TEN (2 × 150 bp read length). The data generated from the Illumina platform were used for bioinformatics analyses as previously described (Barry et al., 2005; Gushgari-Doyle and Alvarez-Cohen, 2020). Statistical significance of differentially expressed transcripts was defined as fold-change ≥ 2 and *p*-value < 0.05. Genes with differential expression were categorized by the Gene Ontology annotation module.

## Results and Discussion

### Enhancement of Trichloroethene-Dechlorination Activity by Iron Sulfide

**FIGURE 1:**
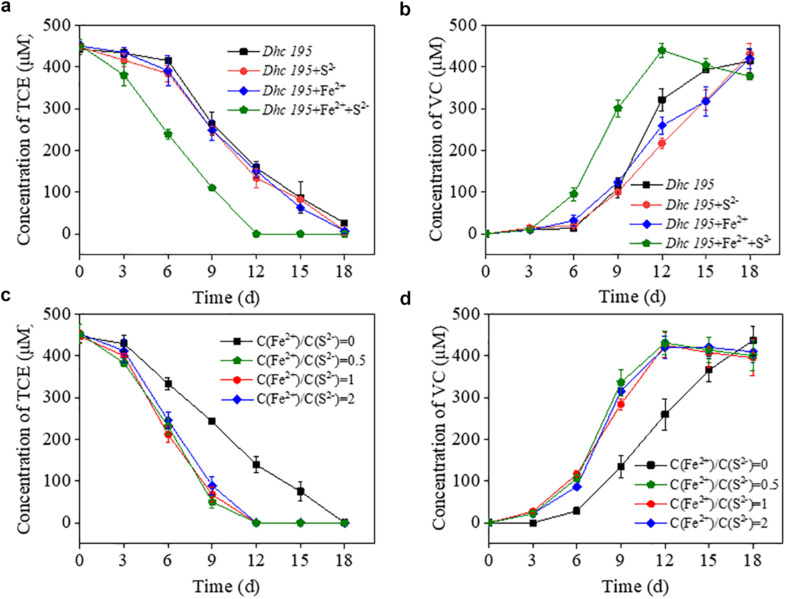
The effects of Fe^2+^ or S^2–^ on the TCE dechlorination by *Dhc* 195 **(a,b)**; The effects of different concentration ratios of Fe^2+^ and S^2–^ on the TCE dechlorination by *Dhc* 195 **(c,d)**.

The initial concentration of S^2–^ (0.2 mM, without adding Fe^2+^) in this study was consistent with the conventional concentration in *Dehalococcoides* medium without toxicity (Löffler et al., 2005; Im et al., 2019). After adding Fe^2+^ (from 0.5 to 1.5 mM) to the cultures (Supplementary Figure 1), the dechlorination period of TCE by *Dhc* 195 was shortened by one-third. To determine the enhanced activity caused by Fe^2+^ or iron–sulfur products, the TCE dechlorination by *Dhc* 195 was evaluated with Fe^2+^ or S^2–^ only (Figures 1a,b). Interestingly, the dechlorination rate of TCE was enhanced from 25.46 ± 1.15 to 37.84 ± 1.89 μmol⋅L^–1^⋅day^–1^, only with the coexistence of Fe^2+^ and S^2–^ (both 0.2 mM), suggesting that the iron–sulfur product improved TCE dechlorination.

Fe^2+^ and S^2–^ might generate multiple forms of iron–sulfur compounds, such as FeS and FeS_2_ (Rickard and Luther, 2007; Xu et al., 2019), under different reactant ratios. Figures 1c,d show that the ratios of Fe^2+^/S^2–^ (i.e., 0.5, 1, and 2) had negligible effects on TCE dechlorination rates, possibly attributed to the similar electrical conductivity of different iron–sulfur compounds. For example, both FeS and FeS_2_ could accelerate electron transport on the surface of nanoscale zero-valent iron (Xu et al., 2020). Multiple kinds of iron–sulfur clusters, such as [4Fe-4S] and [3Fe-4S], are also responsible for transferring electrons to metalloproteins (Zanello, 2018).

**FIGURE 2:**
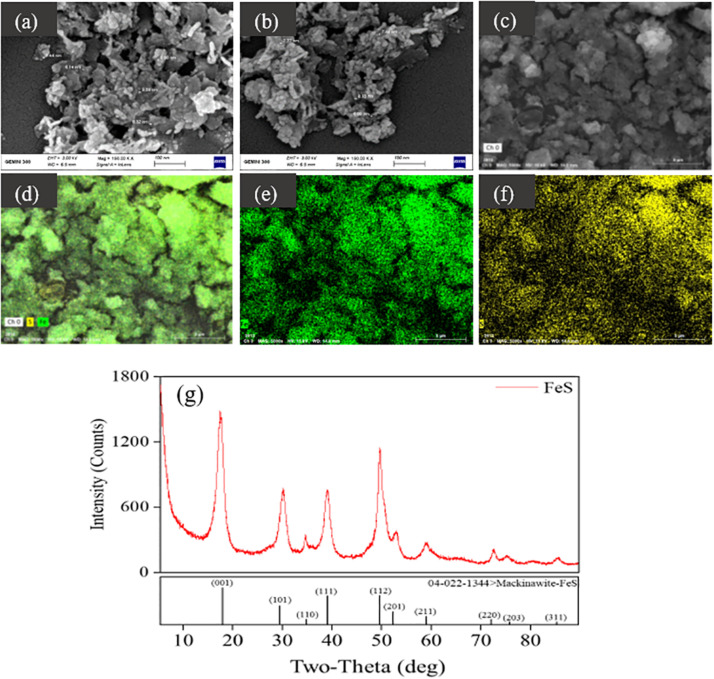
Characterization of formed iron-sulfur nanoparticles with SEM-EDS **(a–f)** and XRD **(g)**.

The exact components of iron–sulfur precipitates produced from Fe^2+^ and S^2–^ (0.2 mM) were revealed by scanning electron microscopy equipped with energy dispersive X-ray spectrometry, showing the uniform distribution of iron and sulfur (Figures 2a–f). The mean size of the formed nanoparticles was smaller than 10 nm, labeled with white lines (Figures 2a,b). Iron–sulfur nanoparticles demonstrated a diffraction pattern identical to FeS (Figure 2g). The crystal structure of naturally formed FeS was similar to the iron–sulfur clusters in *Dhc* 195 (Kublik et al., 2016; Fincker and Spormann, 2017). When Fe^2+^ and S^2–^ entered *Dhc* 195, they may form FeS and affect the activity of *Dhc* 195. Therefore, the optimum concentration of FeS was explored for TCE dechlorination with added concentrations ranging from 0 to 0.6 mM at a Fe^2+^/S^2–^ ratio of 1. The dechlorination rate of TCE reached a maximum at 0.2 mM Fe^2+^ and S^2–^, whereas it was inhibited slightly at 0.6 mM (Supplementary Figure 2). Thus, the best concentration of FeS (0.2 mM) was selected for the downstream experiments.

### Growth Promotion of *Dehalococcoides mccartyi* Strain 195 by Iron Sulfide

**FIGURE 3:**
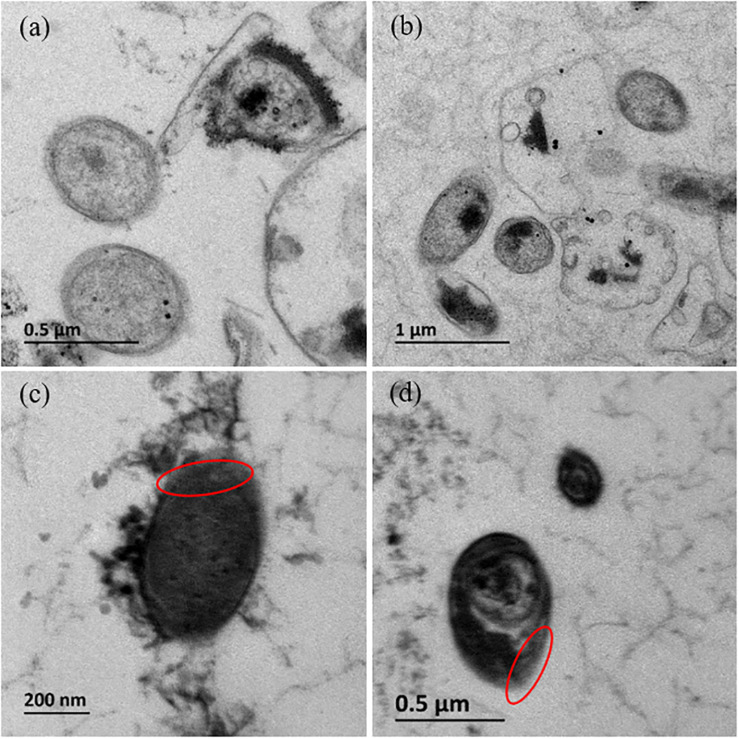
The cellular morphology of *Dhc* 195 (TEM) without FeS **(a,b)** and with FeS **(c,d)**.

**FIGURE 4:**
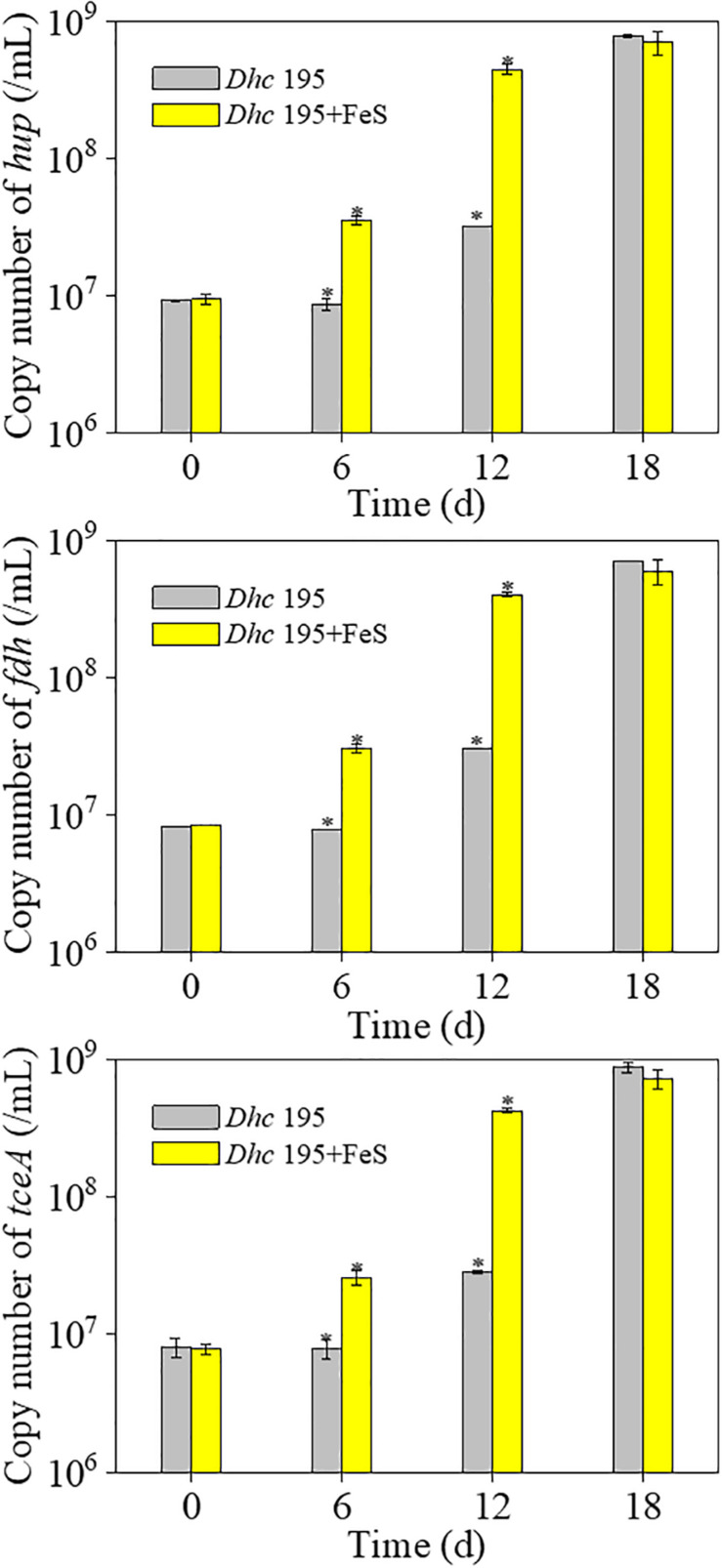
The copy number of three key genes of *Dhe* 195 with and without FeS (^∗^means significant difference, *p*-value < 0.05).

Without the addition of Fe^2+^ and S^2–^, *Dhc* 195 had a regular morphology with a clear cytomembrane, whereas with the addition of Fe^2+^ and S^2–^, many FeS nanoparticles surrounded/entered the cell, and the cytomembrane became misty (Figure 3). The activity of *Dhc* 195 cells should not be destroyed, as FeS enhanced TCE dechlorination but itself could not reduce TCE directly (Jacome et al., 2019). This was consistent with the previous observation, in which 2 mM *in situ* produced FeS did not destroy cells (Deng et al., 2020). Conversely, the growth of *Dhc* 195 was enhanced by the addition of Fe^2+^ and S^2–^. On day 12, the abundance of *tceA* was (2.83 ± 0.13) × 10^7^ copies/ml without FeS, whereas it increased to (4.27 ± 0.21) × 10^8^ copies/ml with FeS (Figure 4). A similar result was also observed in the cell yields from dechlorination, with 6.4 times more cells per molar produced Cl^–^ than the FeS-absent treatment over 12 days (Supplementary Table 3). *Dhc* 195 required hydrogen, instead of Fe^2+^ or S^2–^, as the obligate electron donor for bioreductive dechlorination (Rosenthal et al., 2004; Mao et al., 2015). Therefore, the promoted cell growth of *Dhc* 195 by FeS is probably due to the regulation of physiological activity in *Dhc* 195 (Yan et al., 2013).

### Differential Gene Expression of *Dehalococcoides mccartyi* Strain 195

**FIGURE 5:**
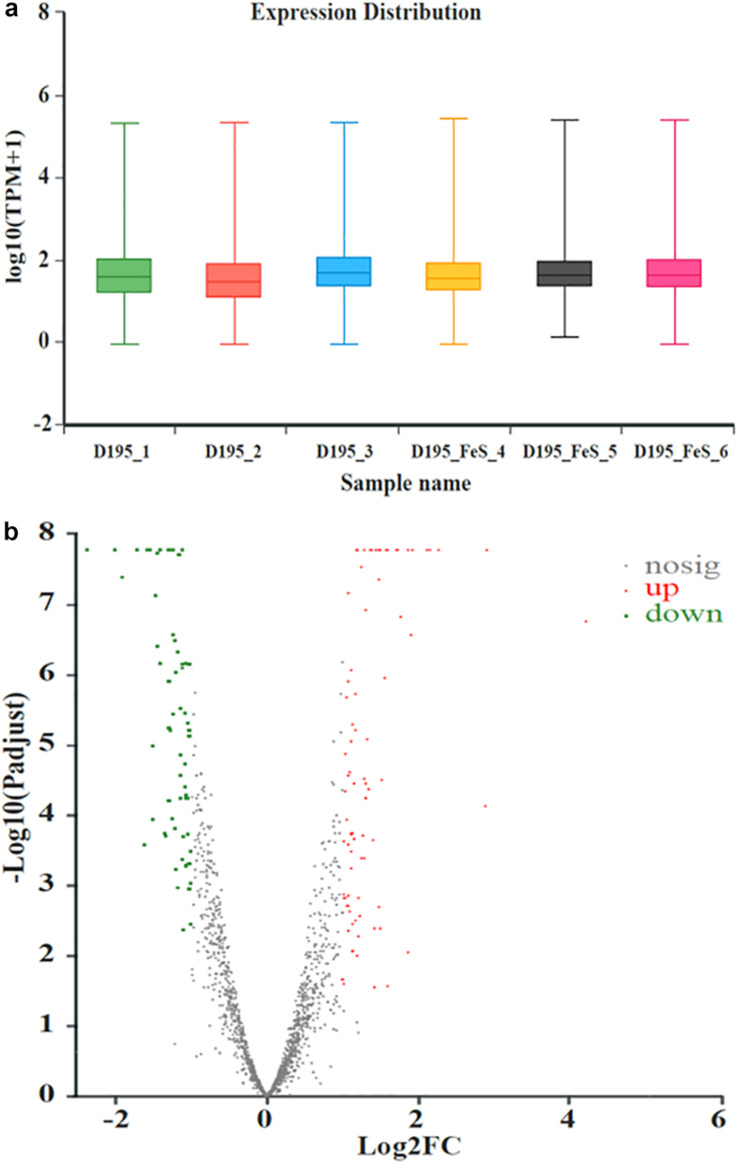
The expression distribution of transcripts for *Dhc* 195 **(a)**; Microarray signal from *Dhc* 195 of two cultures (with and without FeS) **(b)** (≥2-fold difference, *p*-value < 0.05). The red dots represented the up-regulated genes and the green dots represented the down-regulated genes with the addition of FeS. All measurements were avenges from three biological replicates.

**FIGURE 6:**
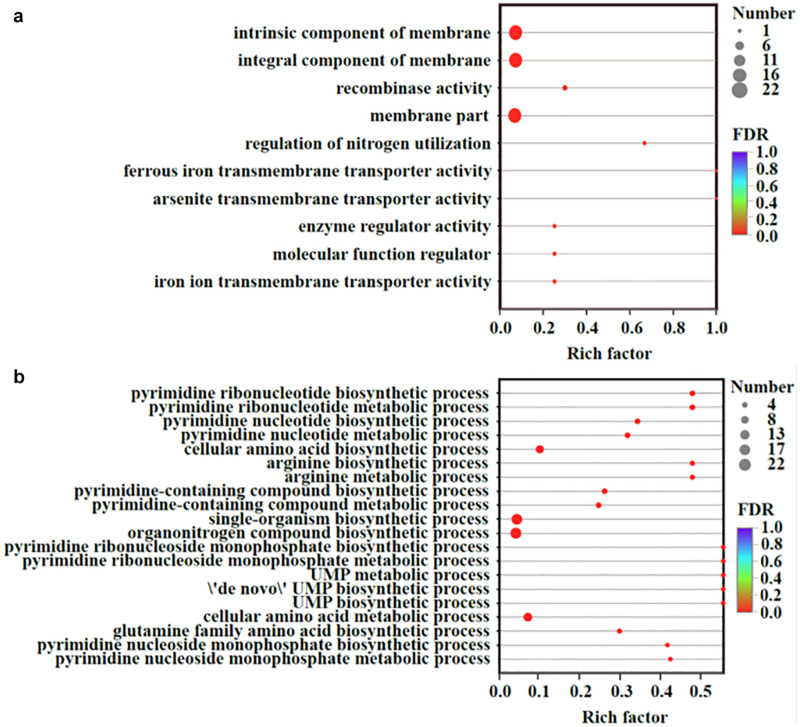
Up-regulated **(a)** and down-regulated **(b)** genes organized by the GO enrichment analysis. Vertical axis represented the GO term; Horizontal axis represented the ratios of Sample number/Background number. The size of dots indicated the number of genes/transcripts in this GO term. All measurements were averages from three biological replicates.

Transcriptomic analysis was performed to identify gene expression after adding Fe^2+^ and S^2–^. The total expression pattern of cultures amended with and without FeS was relatively stable (Figure 5a). In Fe^2+^- and S^2–^-amended cultures, 90 genes were upregulated, and 63 genes were downregulated (≥2-fold change, *p*-value < 0.05) (Figure 5b and Supplementary Tables 4, 5). Among the upregulated genes, the main functions were associated with transmembrane transporter activity, enzyme regulator activity, molecular function regulator, and membrane components (Figure 6a). The addition of FeS enhanced the transcriptomic activity of genes responsible for the transmembrane transport of iron (DET1503, DET0685) and cobalamin (DET0685, DET1139, *cobA*), which is the active center of RDase (Yan et al., 2017). Meanwhile, *modA* (DET1161), involved in molybdenum transport and periplasmic molybdate binding, was upregulated 2.16 times; the gene (DET1632) encoding iron–sulfur cluster assembly protein was upregulated 2.11 times. Given the molybdenum and iron–sulfur clusters as the CISM center and the electron transporter, respectively (Mansfeldt et al., 2014; Kublik et al., 2016), these upregulations together suggested that electron transfer in the ETC was enhanced by the addition of FeS.

The transcriptional repressor gene LexA (DET0274) was also upregulated, resulting in the downregulation of some genes. Some genes (such as DET0657, DET0691, DET1198, and DET1481) were related to the biosynthesis of amino acids and pyrimidine-containing compounds (Figure 6b). They were involved in some processes, such as phosphorylation, secretion, and secondary processes. For example, the gene *ccdA* (DET0619), encoding a cytochrome *c*-type biogenesis protein, was downregulated, but it had little effect on the ETC of *Dhc* 195. Although the *cobT* (DET0657, DET0691) genes participating in cobalamin biosynthesis were downregulated, cobalamin (vitamin B_12_) was extrinsic and added directly to our experiments. The downregulation of *cobT* may have little effect on the activity of *Dhc* 195.

In terms of genes associated with TCE dechlorination in the ETC, only three of 35 putative RDase genes were upregulated, whereas the dominant RDase gene *tceA* (DET 0079) showed no significant differential expression. The stable expression of RDase genes was also reported by Mao et al. (2015), although the yield of *Dhc* 195 increased approximately 16 times when cocultured with *Syntrophomonas wolfei*. The absolute transcription of three key genes, *tceA*, *hup*, and *fdh*, was further quantified at the optimum concentration of FeS (0.2 mM). With the addition of FeS, the transcript number of *tceA* was increased approximately five times on day 12, whereas *hup* and *fdh* were increased approximately three times (Supplementary Figure 3). The increased transcription of key genes confirmed the enhanced electron transport in the ETC for TCE dechlorination.

Transcription is an indicator of the physiological activity of *Dehalococcoides* (Lee et al., 2006; Rahm et al., 2006; Mao et al., 2017). Throughout the transcriptomic results discussed earlier, it was obvious that the addition of Fe^2+^ and S^2–^ upregulated transmembrane transporter genes such as *cobA* and *modA* and iron–sulfur cluster assembly genes. The transcript numbers of key genes, including *tceA*, *hup*, and *fdh*, in the ETC were also increased. This physiological evidence indicated that FeS enhanced the dechlorination of TCE by promoting electron transport in the ETC.

## Conclusion

Bioremediation is a promising method for the *in situ* treatment of polluted soil and groundwater because of its low cost and low secondary pollution (Wang et al., 2016; Zhao et al., 2020). However, the inefficient reduction of organohalides is still a challenge due to the strict growth conditions of *Dehalococcoides*. By adding Fe^2+^ and S^2–^, both the dechlorination rate of TCE and the growth of *Dhc* 195, as well as the abundance of key genes, were improved substantially. In particular, the transcriptomic activity of genes involved in the electron transport chain was enhanced, suggesting that FeS promoted the dechlorination of TCE by regulating the electron transport of *Dhc* 195. Collectively, this study provides an eco-friendly and effective method to increase the dechlorination efficiency of organochlorides. Fe^2+^ and S^2–^ are ubiquitous in the natural environment and are produced by the reduction of Fe^3+^ and SO_4_^2–^ under the activity of iron- and sulfate-reducing bacteria (Trudinger et al., 1985; Flynn et al., 2014; Sheu et al., 2016; Lu et al., 2017; Picard et al., 2019). With the coexistence of organohalide-respiring bacteria, this method has wide application prospects for removing organohalide pollution *in situ*.

## Data Availability Statement

The raw data supporting the conclusions of this article will be made available by the authors, without undue reservation.

## Author Contributions

YL designed, analysis, interpretation, and wrote the manuscript. H-PZ revised the manuscript critically for important intellectual content. LZ participated in conception, design, and approval of the final version. All authors contributed to the article and approved the submitted version.

## Conflict of Interest

The authors declare that the research was conducted in the absence of any commercial or financial relationships that could be construed as a potential conflict of interest.
