# Supplementary figures and images for: Iron Sulfide Enhanced the Dechlorination of Trichloroethene by Dehalococcoides mccartyi Strain 195
Source: Front Microbiol. 2021 Jun 1;12:665281. doi: 10.3389/fmicb.2021.665281 (PMC8203822; doi:10.3389/fmicb.2021.665281)

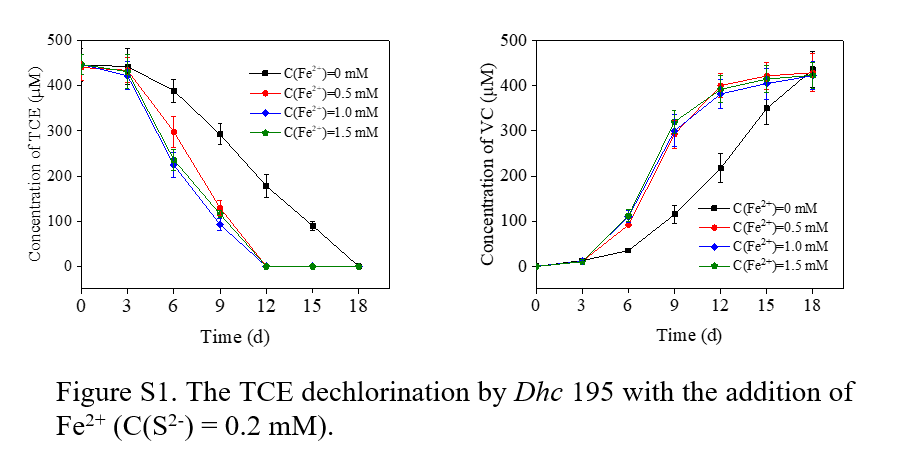

Supplement: Supplementary file 1 [file Image_1.TIF]

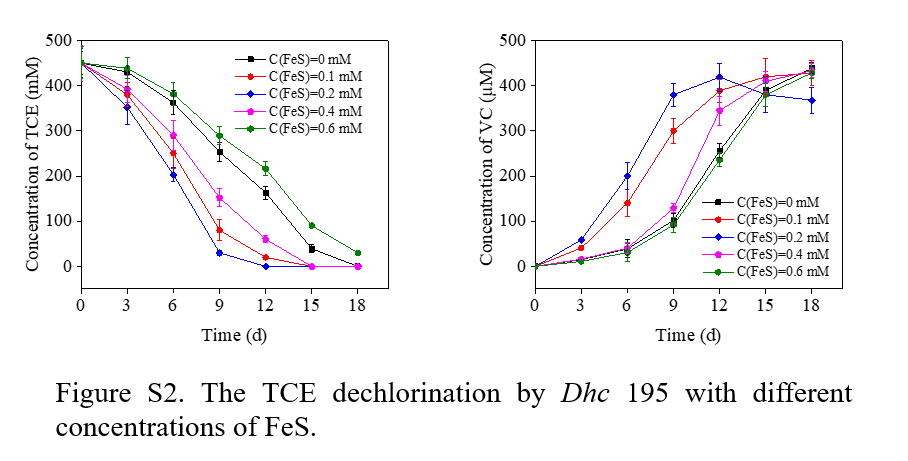

Supplement: Supplementary file 2 [file Image_2.TIF]

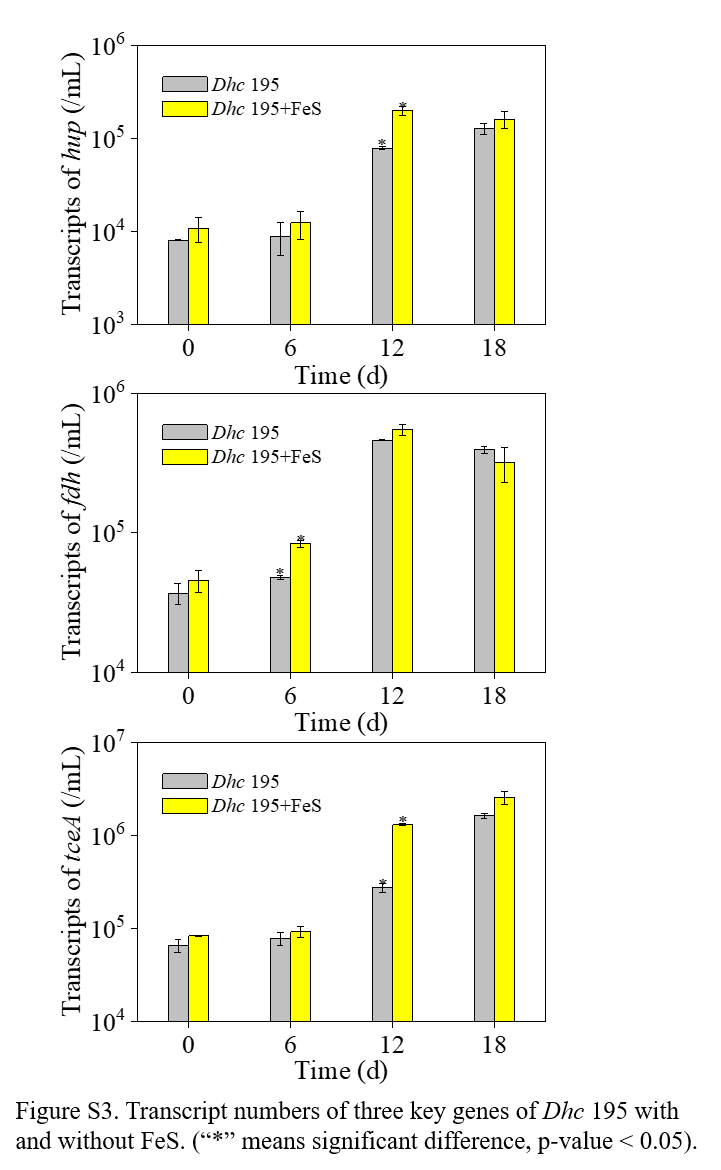

Supplement: Supplementary file 3 [file Image_3.TIF]
