# Supplementary material for: Iron Sulfide Enhanced the Dechlorination of Trichloroethene by Dehalococcoides mccartyi Strain 195
Source: Front Microbiol. 2021 Jun 1;12:665281. doi: 10.3389/fmicb.2021.665281 (PMC8203822; doi:10.3389/fmicb.2021.665281)
Supplement: Supplementary file 4 [file Table_1.DOCX]

Table S1. Primers and probes used for the RT-PCR.

| Primers/probes | Sequence (5’-3’) | Targeted gene | References |
| --- | --- | --- | --- |
| TceA1270F | ATCCAGATTATGACCCTGGTGAA | *tceA* gene of *Dehalococcoides mccartyi* strain 195 | Ritalahti et al., 2006 |
| TceA1336R | GCGGCATATATTAGGGCATCTT |  |  |
| TceA1294Probe | FAM-TGGGCTATGGCGACCGCAGG-TAMRA |  |  |
| HupF | TGACGTTATTGCAGTAGCTGAGT | *hup* gene of *Dehalococcoides mccartyi* strain 195 | Morris et al., 2006 |
| HupR | CACACCATAGCTGAGCAGGTT |  |  |
| FdhF | CCAGACTGAGTACCGCTTCCA | *fdh* gene of *Dehalococcoides mccartyi* strain 195 | Rahm et al., 2006 |
| FdhR | GGAGAAACAGGCGGGGTAGT |  |  |

The TaqMan probe has 6-carboxyfluorescein (FAM) as a reporter fluorophore on the 5’ end, and N,N,N’,N’-tetramethyl-6-carboxyrhodamine (TAMRA) as quencher on the 3’ end.
