# Supplementary material for: Iron Sulfide Enhanced the Dechlorination of Trichloroethene by Dehalococcoides mccartyi Strain 195
Source: Front Microbiol. 2021 Jun 1;12:665281. doi: 10.3389/fmicb.2021.665281 (PMC8203822; doi:10.3389/fmicb.2021.665281)
Supplement: Supplementary file 5 [file Table_2.DOCX]

Table S2. Standard curve of selected genes.

| Genes | Standard curve | R^2^ | Amplification efficiency (%) |
| --- | --- | --- | --- |
| *tceA* | y=-2.641x+38.719 | 0.9985 | 105 |
| *hup* | y=-2.891x+35.119 | 0.9759 | 102 |
| *fdh* | y=-4.175x+43.704 | 0.9952 | 97 |
