# Supplementary material for: Iron Sulfide Enhanced the Dechlorination of Trichloroethene by Dehalococcoides mccartyi Strain 195
Source: Front Microbiol. 2021 Jun 1;12:665281. doi: 10.3389/fmicb.2021.665281 (PMC8203822; doi:10.3389/fmicb.2021.665281)
Supplement: Supplementary file 6 [file Table_3.DOCX]

Table S3. Cell yield of *Dhc* 195 with or without FeS.

| Time/d | *Dhc* 195 (cell/μmol of Cl^-^) | *Dhc* 195 +FeS (cell/μmol of Cl^-^) |
| --- | --- | --- |
| 6 | (9.25±0.81) × 10^6^ | (5.71±0.60) × 10^7^ |
| 12 | (3.71±0.53) × 10^7^ | (2.37±0.41) × 10^8^ |
| 18 | (9.86±1.01) × 10^8^ | (7.34±0.58) × 10^8^ |
