# Supplementary material for: Iron Sulfide Enhanced the Dechlorination of Trichloroethene by Dehalococcoides mccartyi Strain 195
Source: Front Microbiol. 2021 Jun 1;12:665281. doi: 10.3389/fmicb.2021.665281 (PMC8203822; doi:10.3389/fmicb.2021.665281)
Supplement: Supplementary file 7 [file Table_4.DOCX]

Table S4. Significantly up-regulated genes in response to FeS in *Dhc* 195.

| Gene_ID | Gene description | FC(D195_FeS/D195) | p-value |
| --- | --- | --- | --- |
| DET0023 | hypothetical protein | 2.334 | 0.00030602 |
| DET0068 | hypothetical protein | 2.277 | 0.005960194 |
| DET0080 | hypothetical protein | 2.261 | 0.000823775 |
| DET0086 | hipB protein, putative | 2.67 | 0.01781949 |
| DET0107 | hypothetical protein | 2.789 | 0.001137121 |
| DET0134 | hypothetical protein | 2.105 | 0.000358684 |
| DET0174 | lipoprotein, putative | 2.82 | 0.001923897 |
| DET0180 | reductive dehalogenase, putative | 2.001 | 4.35998E-06 |
| DET0256 | hypothetical protein | 2.171 | 2.3359E-05 |
| DET0259 | membrane protein, putative | 2.208 | 0.004605488 |
| DET0275 | hypothetical protein | 2.675 | 0.001993277 |
| DET0282 | membrane protein, putative | 2.208 | 0.004605488 |
| DET0294 | hypothetical protein | 2.481 | 7.0415E-06 |
| DET0313 | hypothetical protein | 2.005 | 0.013261591 |
| DET0319 | reductive dehalogenase anchoring protein, putative | 2.225 | 3.56789E-06 |
| DET0469 | hypothetical protein | 2.037 | 0.000565166 |
| DET0471 | ribosomal protein S7 | 2.642 | 3.30105E-05 |
| DET0476 | ribosomal protein L23 | 3.044 | 0.008703819 |
| DET0498 | ribosomal protein L36 | 7.449 | 6.00335E-12 |
| DET0499 | ribosomal protein S13 | 2.045 | 7.11634E-07 |
| DET0507 | hypothetical protein | 2.137 | 0.000680626 |
| DET0548 | DNA-binding protein | 2.347 | 0.001342753 |
| DET0634 | hypothetical protein | 2.168 | 8.29709E-07 |
| DET0685 | ABC-type cobalamin/Fe^3+^-siderophores transport systems, permease component | 3.731 | 1.80706E-08 |
| DET0751 | ribosomal protein L35 | 2.876 | 3.25531E-06 |
| DET0768 | conserved hypothetical protein | 2.328 | 0.004635589 |
| DET0880 | transcriptional regulator, LuxR family | 2.382 | 0.000109979 |
| DET0892 | membrane protein, putative | 2.208 | 0.004605488 |
| DET0904 | hypothetical protein | 2.481 | 7.0415E-06 |
| DET0908 | arsenical pump membrane protein, putative | 2.023 | 0.000347801 |
| DET0954 | Hsp20/alpha crystallin family protein | 2.812 | 1.06663E-09 |
| DET0959 | Smr domain protein | 2.051 | 0.000487775 |
| DET1021 | hypothetical protein | 4.319 | 2.58073E-10 |
| DET1057 | conserved hypothetical protein | 18.471 | 5.68897E-09 |
| DET1058 | DNA-binding response regulator | 2.578 | 2.62759E-12 |
| DET1067 | site-specific recombinase, phage integrase family | 2.391 | 7.68041E-10 |
| DET1068 | site-specific recombinase, phage integrase family, truncation | 2.254 | 2.08977E-07 |
| DET1069 | site-specific recombinase, phage integrase family | 2.455 | 3.40442E-10 |
| DET1071 | holin | 2.473 | 8.84698E-09 |
| Continued table | |  |  |
| DET1072 | hypothetical protein | 3.779 | 4.14275E-23 |
| DET1074 | hypothetical protein | 2.602 | 7.53746E-13 |
| DET1075 | hypothetical protein | 2.185 | 3.04829E-07 |
| DET1076 | hypothetical protein | 2.291 | 1.76255E-11 |
| DET1077 | hypothetical protein | 2.707 | 7.85495E-12 |
| DET1078 | tail tape measure protein, TP901 family | 2.978 | 7.23842E-19 |
| DET1079 | conserved hypothetical protein | 2.518 | 6.59759E-07 |
| DET1080 | major tail protein, phi13 family | 2.252 | 4.90868E-07 |
| DET1081 | conserved hypothetical protein | 3.32 | 2.39056E-10 |
| DET1082 | conserved hypothetical protein | 3.032 | 1.32338E-11 |
| DET1083 | head-tail adaptor, putative | 2.062 | 5.9001E-06 |
| DET1085 | major capsid protein, HK97 family | 2.289 | 3.71723E-11 |
| DET1087 | portal protein, HK97 family, putative | 2.816 | 1.41511E-12 |
| DET1088 | terminase, large subunit, putative | 2.273 | 1.0904E-10 |
| DET1089 | virulence-related protein | 4.46 | 9.54216E-25 |
| DET1090 | hypothetical protein | 3.423 | 2.85631E-08 |
| DET1091 | virulence-related protein | 2.732 | 4.71435E-18 |
| DET1092 | DNA methylase | 2.119 | 1.6763E-06 |
| DET1093 | hypothetical protein | 3.67 | 2.75073E-21 |
| DET1096 | SNF2 domain protein | 2.295 | 8.46504E-13 |
| DET1097 | conserved hypothetical protein | 2.791 | 2.28183E-11 |
| DET1099 | hypothetical protein | 3.281 | 4.46128E-14 |
| DET1103 | hypothetical protein | 2.115 | 3.68043E-08 |
| DET1104 | hypothetical protein | 2.477 | 3.75684E-06 |
| DET1106 | hypothetical protein | 2.234 | 4.41248E-05 |
| DET1113 | conserved domain protein | 2.079 | 0.00056796 |
| DET1139 | cob(I)alamin adenosyltransferase | 2.172 | 0.000120163 |
| DET1156 | nitrogen regulatory protein P-II | 2.122 | 0.000785746 |
| DET1157 | nitrogen regulatory protein P-II | 2.013 | 0.014851774 |
| DET1161 | molybdenum ABC transporter, periplasmic molybdate-binding protein | 2.163 | 3.84622E-05 |
| DET1166 | hypothetical protein | 2.005 | 0.013261591 |
| DET1169 | hypothetical protein | 2.199 | 0.001811 |
| DET1220 | hypothetical protein | 2.533 | 7.46333E-06 |
| DET1319 | hypothetical protein | 2.417 | 3.61628E-05 |
| DET1324 | conserved hypothetical protein | 2.437 | 7.229E-05 |
| DET1348 | RNA polymerase sigma-70 factor, ECF subfamily | 2.081 | 9.67514E-06 |
| DET1369 | hypothetical protein | 2.448 | 3.85961E-06 |
| DET1376 | conserved hypothetical protein | 2.125 | 1.91965E-06 |
| DET1384 | preprotein translocase, YajC subunit | 3.67 | 0.002200946 |
| DET1443 | hypothetical protein | 2.16 | 7.59681E-05 |
| DET1496 | conserved hypothetical protein | 2.948 | 2.07693E-07 |
| DET1503 | ferrous iron transport protein B, putative | 2.015 | 2.73561E-05 |
| Continued table | |  |  |
| DET1537 | reductive dehalogenase anchoring protein, putative | 2.099 | 0.002039231 |
| DET1553 | hypothetical protein | 2.168 | 2.84863E-08 |
| DET1568 | hypothetical protein | 7.505 | 3.51732E-27 |
| DET1569 | hypothetical protein | 4.797 | 4.58336E-12 |
| DET1582 | conserved domain protein | 2.08 | 5.48604E-08 |
| DET1584 | hypothetical protein | 2.181 | 4.35745E-05 |
| DET1597 | hypothetical protein | 2.32 | 0.002925103 |
| DET1623 | desulforedoxin, putative | 2.112 | 5.68408E-05 |
| DET1632 | NifU-like protein | 2.11 | 1.17827E-09 |
