# Supplementary material for: Iron Sulfide Enhanced the Dechlorination of Trichloroethene by Dehalococcoides mccartyi Strain 195
Source: Front Microbiol. 2021 Jun 1;12:665281. doi: 10.3389/fmicb.2021.665281 (PMC8203822; doi:10.3389/fmicb.2021.665281)
Supplement: Supplementary file 8 [file Table_5.DOCX]

Table S5. Significantly down-regulated genes in response to FeS in *Dhc* 195.

| Gene_ID | Gene description | FC(D195_FeS/D195) | p-value |
| --- | --- | --- | --- |
| DET0001 | chromosomal replication initiator protein DnaA | 0.5 | 4.20751E-05 |
| DET0111 | [Ni/Fe] hydrogenase, group 1, small subunit, putative | 0.444 | 0.000216692 |
| DET0114 | ABC transporter, substrate-binding protein, putative | 0.476 | 0.000100845 |
| DET0142 | phosphate transport system regulatory protein PhoU | 0.49 | 4.36218E-07 |
| DET0186 | formate dehydrogenase, membrane subunit, putative | 0.353 | 8.81976E-07 |
| DET0351 | hypothetical protein | 0.394 | 5.87506E-05 |
| DET0358 | hypothetical protein | 0.365 | 1.93434E-08 |
| DET0372 | phosphatidate cytidylyltransferase | 0.481 | 0.000114637 |
| DET0378 | phosphoribosylformylglycinamidine synthase I | 0.443 | 1.30844E-08 |
| DET0436 | Serine hydroxymethyltransferase | 0.457 | 8.40817E-07 |
| DET0451 | malate dehydrogenase, NAD-dependent | 0.483 | 5.155E-06 |
| DET0463 | 3-phosphoshikimate 1-carboxyvinyltransferase | 0.457 | 7.01909E-05 |
| DET0465 | shikimate 5-dehydrogenase | 0.488 | 4.79926E-06 |
| DET0466 | 3-dehydroquinate dehydratase, type I | 0.431 | 2.19466E-05 |
| DET0553 | Deoxyguanosinetriphosphate triphosphohydrolase, putative | 0.304 | 2.53158E-11 |
| DET0592 | hypothetical protein | 0.433 | 0.146912302 |
| DET0596 | DNA ligase, NAD-dependent | 0.473 | 1.11254E-06 |
| DET0599 | D-3-phosphoglycerate dehydrogenase | 0.437 | 3.47372E-08 |
| DET0604 | DNA-directed RNA polymerase, beta' subunit | 0.467 | 2.54163E-05 |
| DET0619 | cytochrome c-type biogenesis protein CcdA | 0.414 | 3.99789E-07 |
| DET0624 | response regulator | 0.426 | 6.84056E-12 |
| DET0636 | cell division protein FtsZ | 0.482 | 4.20656E-06 |
| DET0649 | hypothetical protein | 0.177 | 0.139885979 |
| DET0657 | nicotinate-nucleotide-dimethylbenzimidazole phosphoribosyltransferase | 0.193 | 3.26707E-18 |
| DET0691 | nicotinate-nucleotide--dimethylbenzimidazole phosphoribosyltransferase | 0.193 | 3.26707E-18 |
| DET0715 | metallopeptidase, M24 family | 0.25 | 4.05303E-13 |
| DET0754 | hypothetical protein | 0.499 | 0.000876199 |
| DET0766 | V-type H(+)-translocating pyrophosphatase | 0.374 | 3.3907E-08 |
| DET0845 | ATP phosphoribosyltransferase, putaitve | 0.423 | 8.73523E-09 |
| DET0871 | transcriptional regulator, MarR family | 0.406 | 4.29509E-07 |
| DET0872 | membrane protein, MmpL family | 0.462 | 1.26286E-13 |
| DET0926 | proton-translocating NADH-quinone oxidoreductase, D subunit | 0.471 | 3.47065E-06 |
| DET0928 | proton-translocating NADH-quinone oxidoreductase, I subunit | 0.488 | 2.86234E-07 |
| DET0980 | hypothetical protein | 0.324 | 6.5901E-05 |
| DET1034 | hypothetical protein | 0.453 | 1.3994E-07 |
| DET1035 | pyridoxal-phosphate dependent TrpB-like enzyme | 0.493 | 7.24611E-05 |
| Continued table | |  |  |
| DET1146 | hypothetical protein | 0.353 | 3.74551E-05 |
| DET1198 | pyrimidine operon regulatory protein/uracil phosphoribosyl transferase | 0.408 | 6.21226E-08 |
| DET1199 | aspartate carbamoyl transferase | 0.471 | 1.32284E-07 |
| DET1200 | dihydroorotase, multifunctional complex type | 0.36 | 2.57053E-09 |
| DET1201 | carbamoyl-phosphate synthase, small subunit | 0.432 | 8.61853E-09 |
| DET1202 | carbamoyl-phosphate synthase, large subunit | 0.408 | 1.40473E-11 |
| DET1203 | dihydroorotate dehydrogenase, electron transfer subunit | 0.267 | 2.10064E-09 |
| DET1205 | threonine synthase | 0.457 | 2.77058E-08 |
| DET1257 | acetylglutamate kinase | 0.456 | 5.64142E-06 |
| DET1260 | argininosuccinate synthase | 0.332 | 4.32227E-11 |
| DET1261 | argininosuccinate lyase | 0.459 | 1.96309E-08 |
| DET1275 | ribosomal protein L32 | 0.468 | 0.00129277 |
| DET1280 | radical SAM/B12 binding domain protein | 0.45 | 2.58692E-10 |
| DET1335 | glutamyl-tRNA (Gln) amidotransferase, A subunit | 0.376 | 9.48839E-12 |
| DET1359 | twitching mobility protein | 0.494 | 2.43256E-07 |
| DET1360 | general secretion family protein | 0.344 | 1.17427E-11 |
| DET1364 | conserved hypothetical protein | 0.486 | 2.72337E-05 |
| DET1393 | hypothetical protein | 0.397 | 3.15272E-05 |
| DET1399 | chaperone protein DnaK | 0.453 | 1.73434E-06 |
| DET1407 | BNR/Asp-box repeat domain protein | 0.438 | 0.000111079 |
| DET1418 | nicotinate-nucleotide pyrophosphorylase, putative | 0.425 | 2.32363E-07 |
| DET1435 | hydrogenase expression/formation protein HypE | 0.478 | 5.07505E-06 |
| DET1463 | hypothetical protein | 0.498 | 0.000218498 |
| DET1532 | dinitrogenase iron-molybdenum cofactor family protein | 0.421 | 1.41697E-05 |
| DET1608 | KH domain/HDIG domain protein | 0.479 | 1.59638E-08 |
| DET1609 | Ser/Thr protein phosphatase family protein | 0.489 | 1.8396E-08 |
| DET1621 | hypothetical protein | 0.369 | 4.98087E-10 |
